# The Role of Protein Kinase CK2 in Development and Disease Progression: A Critical Review

**DOI:** 10.3390/jdb10030031

**Published:** 2022-07-27

**Authors:** Daniel Halloran, Venu Pandit, Anja Nohe

**Affiliations:** Department of Biological Sciences, University of Delaware, Newark, DE 19716, USA; dhallor@udel.edu (D.H.); vpandit@udel.edu (V.P.)

**Keywords:** casein kinase II, development, cancer, CX-4945, CIBG-300

## Abstract

Protein kinase CK2 (CK2) is a ubiquitous holoenzyme involved in a wide array of developmental processes. The involvement of CK2 in events such as neurogenesis, cardiogenesis, skeletogenesis, and spermatogenesis is essential for the viability of almost all organisms, and its role has been conserved throughout evolution. Further into adulthood, CK2 continues to function as a key regulator of pathways affecting crucial processes such as osteogenesis, adipogenesis, chondrogenesis, neuron differentiation, and the immune response. Due to its vast role in a multitude of pathways, aberrant functioning of this kinase leads to embryonic lethality and numerous diseases and disorders, including cancer and neurological disorders. As a result, CK2 is a popular target for interventions aiming to treat the aforementioned diseases. Specifically, two CK2 inhibitors, namely CX-4945 and CIBG-300, are in the early stages of clinical testing and exhibit promise for treating cancer and other disorders. Further, other researchers around the world are focusing on CK2 to treat bone disorders. This review summarizes the current understanding of CK2 in development, the structure of CK2, the targets and signaling pathways of CK2, the implication of CK2 in disease progression, and the recent therapeutics developed to inhibit the dysregulation of CK2 function in various diseases.

## 1. Introduction

During development, the phosphorylation and dephosphorylation of proteins is essential for the proper formation of organisms [1,2,3]. This process is catalyzed primarily by enzymes that function as kinases, phosphatases, or both [4,5]. Currently, over 530 protein kinases have been identified in humans, many of them involved in embryonic and developmental processes [6,7,8,9]. One of the proteins, protein kinase CK2 (CK2), formerly known as casein kinase II, regulates many of these processes and is expressed during embryogenesis and adulthood [10,11]. CK2 is a holoenzyme consisting of two catalytic subunits (two CK2α, CK2α’, or one CK2α and one CK2α’) and two regulatory beta subunits (CK2β) [12,13,14]. This kinase has over 200 substrates and targets more than 280 phosphorylation sites, indicating its ubiquitous expression and involvement in many cell signaling pathways and molecular processes [10]. It is present in every eukaryotic cell and has been highly conserved throughout evolution. CK2 regulates developmental pathways such as neurogenesis and cardiogenesis, as well as processes in adulthood such as chondrogenesis, osteogenesis, and adipogenesis [15,16,17,18,19,20,21,22]. Further, CK2 is implicated in the progression of diseases including cancer, Alzheimer’s disease, senescence, diabetes, obesity, infectious disease, and cardiovascular diseases [23,24,25]. Due to the vast involvement of CK2 in embryogenesis, development, adulthood, and disease progression, this review aims to summarize the current understanding of this enzyme.

## 2. CK2 and Development

**Figure 1:**
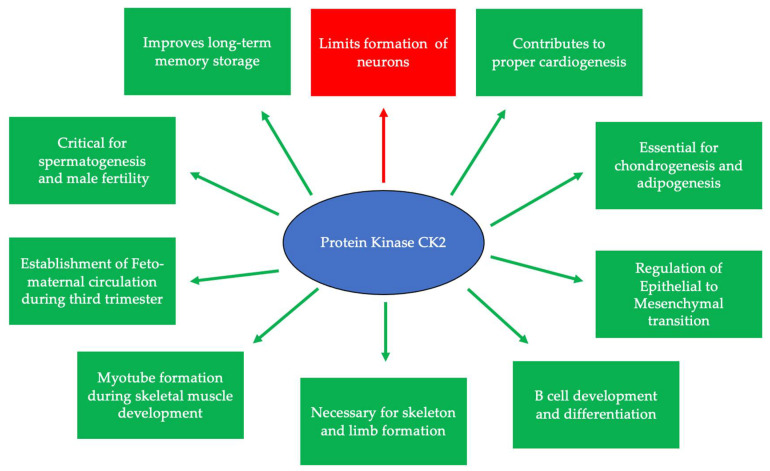
CK2 is expressed at embryonic day 11 and is critical for many developmental processes. Specifically, CK2 is necessary for limiting neurogenesis and preventing the excessive differentiation of neurons. In addition, CK2 expression promotes long-term memory storage. Further, this protein is essential for skeletogenesis, chondrogenesis, adipogenesis, and proper limb formation. CK2 contributes to spermatogenesis, and the inhibition of its expression leads to infertility. Finally, CK2 is important for B cell differentiation and development, myotube formation, the regulation of the epithelial-to-mesenchymal transition (EMT), and the establishment of circulation between the fetus and the mother during the third trimester.

CK2 is highly expressed and prominent throughout many stages of development. During early embryogenesis, CK2 activity was identified first at embryonic day 11 (E11) in mice, rats, and chickens [26,27,28]. Continuously after this day, CK2 regulates the formation of various organ systems. Specifically, during neurogenesis and regarding the negative regulation of neuron differentiation, CK2 phosphorylates Groucho/Transducin-like enhancer (TLE1) to limit the formation of neurons [29]. CK2α null mice display improper neural tube formation, emphasizing the essential role of CK2 in this process [30]. Furthermore, CK2 contributes to memory formation, and the overexpression of this kinase is correlated with improved long-term memory [31]. In cardiogenesis, CK2, specifically CK2α’, is essential for the proper formation of the heart, and knockouts of this subunit lead to irregular cardiogenesis, as well as death in mid-gestation [30]. Similarly, CK2 is critical for proper spermatogenesis and the fertility of organisms [32]. While CK2α’^−/−^ mice remain viable, male mice are infertile and incapable of producing offspring, as germ cells do not form properly [30,33,34]. Moreover, CK2α’ is highly expressed in testis, and a lack of CK2α’ also leads to oligozoospermia [33]. CK2 is also required for the formation of the skeleton during development, specifically at E17.5 [35]. It has also been demonstrated that the conditional deletion of the gene encoding beta subunit of CK2 (*Csnk2b*) leads to the shortening of the limbs, improper endochondral ossification, and lethality in mice [35]. Therefore, CK2 is critical for many stages of development and the production of viable organisms (Figure 1). The evolutionary conservation of this enzyme is also evident from the effects of its knockout mutations in yeast (Table 1) [36]. In addition, during the third trimester of pregnancy, feto–maternal circulation is established through a mechanism involving trophoblast invasion. This process requires mitochondrial modifications, and CK2 is critical throughout the process. Therefore, this kinase is one of the central enzymes during placental development [37].

**Figure 2:**
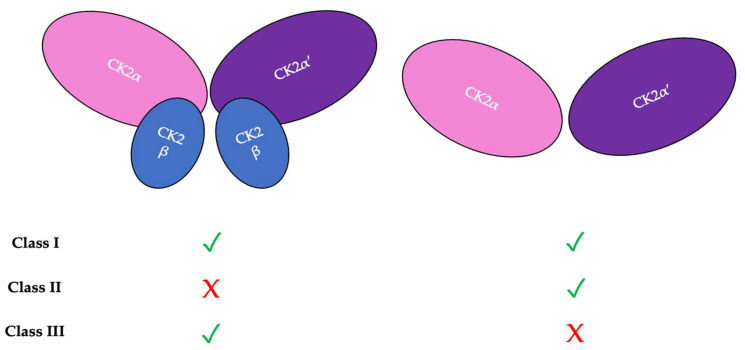
There are three classifications of CK2 substrates. Class I substrates are identified as proteins that are equally phosphorylated by the holoenzyme and individually by the catalytic subunits. Class II substrates are specifically phosphorylated by the catalytic submits of CK2 but not by the holoenzyme. Class III substrates are preferentially targeted and phosphorylated by the holoenzyme but not by the catalytic subunits of CK2.

There are three classifications of substrates for CK2. Class I substrates are equally phosphorylated by the holoenzyme and free catalytic subunits, whereas Class II substrates are phosphorylated by only free catalytic subunits but not by the holoenzyme. Further, substrates of Class III are phosphorylated preferentially by the holoenzyme but not by free catalytic subunits. Thus, the phosphorylation of these different classes varies during development (Figure 2) [45]. To study the role of the individual subunits during development, specific subunit knockouts were induced. As described in Table 1, the involvement of specific subunits in developmental processes is evident from the conditional knockouts and the resulting associated phenotype or molecular consequence (Table 1). Specifically, CK2β knockouts in mice display developmental arrest after implantation [46]. Furthermore, the balance between CK2α and CK2β subunits is also important. The ratio of CK2α:CK2β is critical for maintaining epithelial morphology [47].

## 3. The Substrates and Signaling Pathways Regulated by CK2

The implication of CK2 in many developmental processes is due to its multifunctionality. Specifically, over 400 substates are identified as targets of CK2 [19,21]. While these substrates are involved in a myriad of pathways, some are critical for the survival and maintenance of cells. A notable pathway of the CK2 is Wnt-signaling, as it binds directly to both β-catenin and T cell-specific transcription factor/lymphoid enhancer-binding factor (TCF/LEF) [17,48,49,50,51]. Furthermore, CK2 phosphorylates other substrates such as dishevelled (Dvl) and adenomatous polyposis coli (APC) [17,52,53]. Regarding β-catenin, CK2 phosphorylates this protein at threonine 393, which interacts with Axin to stabilize β-catenin and prevent it being marked for destruction by protein kinase CK1 [54,55].

A second binding site of CK2 is the bone morphogenetic protein receptor type Ia (BMPRIa), which regulates Smad and non-Smad signaling pathways. Within cells, such as osteoblasts, chondrocytes, and adipocytes, CK2 typically binds to BMPRIa and prevents the downstream activation of Smad signaling [56,57,58,59,60,61]. However, once members of the transforming growth factor beta (TGFβ) superfamily, i.e., BMP-2 and BMP-4, bind to BMPRIa, CK2 is released from BMPRIa, leading to the increased differentiation and activity in osteoblasts, osteoclasts, adipocytes, and chondrocytes [18,19,22,58,62,63,64,65,66,67,68,69,70,71,72,73,74].

Aside from Wnts and Bone morphogenetic proteins (BMPs), CK2 is involved in signaling pathways responsible for proliferation and survival, such as Akt/Phosphatidylinositol-3-Kinase (PI3K) signaling. Specifically, Akt is phosphorylated by CK2 at serine 129, which promotes cell viability and proliferation [75,76,77]. The activation of Akt/PI3K by CK2 has been well documented in cancer progression, which will be discussed later in this review. Similarly, CK2 directly interacts with extracellular signal-regulated kinase (Erk) 1/2, thereby activating the mitogen-activated protein (MAP) kinase signaling pathway. After the phosphorylation of Erk1/2 by CK2 at serine residues within the nuclear translocation signal (NTS) region, Erk1/2 is imported into the nucleus by importin7 to function as a transcription factor in promoting cellular processes [78,79,80,81,82].

Next, CK2 activates the nuclear factor kappa B (NF-κB) signaling pathways. In this context, CK2 phosphorylates the RelA/p65 subunit of NF-κB at serine 529, leading to the activation of this protein [83,84,85]. Further, as CK2 phosphorylates NF-κB inhibitor alpha (IκBα) at its C-terminal sites; this leads to increased cellular proliferation and protection against ultraviolet (UV) radiation and has been implicated in cancer progression [86,87,88,89].

In addition, CK2 activates the Janus Kinase/signal transducer and activator of transcription (JAK/STAT) pathway, leading to enhanced cellular survival, differentiation, and proliferation [90]. Here, CK2 binds to the JAKs, which subsequently activate STATs, such as STAT3, to initiate downstream signaling [91,92]. Additionally, CK2 is essential for the activation of the JAK/STAT pathway by interleukins (ILs), such as IL-6, to initiate transient signaling in the immune system [93,94,95].

CK2 also has a critical role in mammalian target of rapamycin (mTOR) signaling. Specifically, the inhibition of CK2 expression is associated with an increased activity of Ikaros, which binds to the mTOR gene to suppress its activity and prevent tumor formation [96,97,98,99,100]. However, the overexpression of CK2 subsequently increases mTOR signaling, leading to poor outcomes such as tumorigenesis and cancer progression [101,102,103,104]. Therefore, by targeting CK2 activity with inhibitors such as CX-4945 (described later in this review), the mTOR pathway can be diminished, leading to improved prognoses in patients.

Furthermore, CK2 plays a role in signaling pathways governing the DNA damage and the repair response. Specifically, CK2 responds to DNA damage and increases the repair response to accelerate the process [105,106]. Here, CK2 phosphorylates X-ray repair, cross complementing protein 4 (XRCC4), XRCC1, DNA-dependent protein kinase, and apurinic/apyrimidinic endonuclease (APE), among others [107,108,109]. Its ability to bind to a multitude of substrates during DNA damage repair aids in the survival and viability of cells.

Finally, an additional pathway in which CK2 is interestingly involved includes zinc signaling. As zinc can serve as a secondary messenger, it is involved in various signaling pathways. Specifically, CK2 can regulate the concentration of zinc through the phosphorylation of one of its channels, zinc channel ZIP7, located on the Golgi apparatus and endoplasmic reticulum (ER) [110,111]. Once this channel is phosphorylated, there is an influx of zinc into the cytoplasm that leads to continued epidermal growth factor receptor (EGFR) signaling and activation of other proteins, such as Erk1/2 [110,111,112]. While the precise mechanism of CK2 and zinc signaling is not fully delineated, targeting this pathway for future therapeutics may be promising. These pathways are summarized precisely and effectively in the publication written by Borgo et al. [106]. Taken together, CK2 is largely involved in the regulation of multiple cellular signaling pathways, cell survival, and regulation at transcriptional and translational levels.

## 4. CK2 Structure and Kinase Activity

To understand the multifunctionality of CK2, its structure and kinase activity must be observed. CK2 is comprised of α and β subunits, both contributing to the activity of this kinase. This enzyme contains 391 amino acids with a molecular mass of 45.1 kDa [113,114,115]. The formation of a heterotetrametric complex contributes to the stability and activity of CK2 [116]. Specifically, the formation of the CK2 holoenzyme may take the form of α_2_β_2_, αα’β_2_, or α’_2_β_2_ subunits [117,118,119]. The interaction of the regulatory and catalytic subunits contributes to the function and enzymatic activity of CK2. In 2001, Niefind et al. crystallized CK2 to determine the precise molecular spatial localizations of its subunits [113]. Here, the β_2_ dimer interacts slightly with the α subunits to connect them and stabilize the molecule [113]. Similarly, the β_2_ subunits interact strongly, whereas the α subunits do not interact at all. This strong interaction is attributed to the formation of salt bridges and hydrogen bonding, the zinc (Zn^2+^) binding motif, and a hydrophobic core [113]. Further, the active site is between the C-terminus, which is helical, and the N-terminus, which has the architecture of many β-sheets [113]. The structure of this active site aids in CK2′s ability to bind to a myriad of substrates and maintain its catalytic activity. When targeting substrates, CK2 typically phosphorylates serine/threonine residues that share the S/T-X-X-D/E/pS/pY consensus sequence [120]. Further, in other circumstances, CK2 can phosphorylate substrates that do not share the consensus sequence, such as serine 392 of p53 or the tyrosine residues of Fpr3 in yeast [120,121,122]. Due to the versatility of this enzyme, CK2 activates numerous interacting proteins, such as nicotinamide adenine dinucleotide phosphate (NADPH) oxidase 1 and transient receptor potential cation channel subfamily M (TRPM) 3 ion channels [123,124]. As CK2 targets many substrates, it has increasingly become a candidate for novel therapeutics, as it is implicated in the progression of many diseases, such as breast and colon cancer [23,125,126,127].

## 5. CK2 Expression and the Progression of Diseases

The ubiquitous expression of CK2 occurs within almost all cellular compartments [128]. The continued identification of its newer substrates broadens the understanding of the processes in which this protein is involved [129]. Alternative forms of this holoenzyme, such as single catalytic or regulatory subunits, have differential expression and functions within tissues. The CK2α’ subunit has enhanced mRNA expression in the testis, skin, and across brain tissue. Further, the CKα and CK2β subunits have a more ubiquitous expression within tissues [130]. Oligomeric super-structures of the CK2 holoenzyme also exist, in which this protein controls the exposure of the catalytic active sites and subsequent enzymatic activity [131]. The balance between the number of catalytic CK2α and regulatory CK2β subunits is maintained by a mechanism whereby CK2α activates the transcription factors for CK2β when its levels are depleted. Once sufficient levels of CK2β are obtained, transcription is inhibited by holoenzyme formation, which then disables the interaction between CK2α and its own transcription factors [132].

Several micro ribonucleic acids (miRNAs) are trans-regulating elements of CK2 protein expression. Small noncoding RNAs targeting CK2 include miR-760, miR-186, miR-337-3p, and miR-216b. These miRNAs reduce CK2α protein expression by binding at the 3′ untranslated region (UTR) of the transcript and inhibiting translation [117,133,134]. During senescence, miR-186, miR-216b, miR-337-3p, and miR-760 have been shown to target the CK2α subunit at the 3′UTR region and cause its degradation [135,136]. Long noncoding RNA KCNQ10T1 and its interaction with miR-760 is a positive regulator of CK2 to enhance its activity [137].

Protein interactors of CK2 also regulate its expression and activity. In chondrocytes, the downregulation of αB-crystallin by CK2 is shown to protect cells against apoptosis. The pharmacological inhibition of CK2 also cause changes in the localization of this protein [138]. Further, HSP90 interacts with CK2 to prevent its self-aggregation and loss of activity [139].

### 5.1. Dynamic Localization of CK2

**Figure 3:**
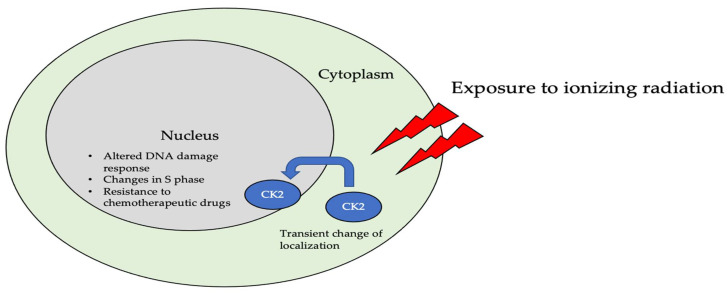
The localization of CK2 changes when cells are exposed to ionizing radiation (IR). Specifically, CK2α is translocated from the cytoplasm to the nucleus in multiple cell lines, including A549, H460, PC9, and M059K. Further, upon radiation exposure, the overall kinase activity of CK2 increases, emphasizing its role in the DNA-damage-repair response, as it colocalizes with DNA in the nucleus of cells.

**Figure 4:**
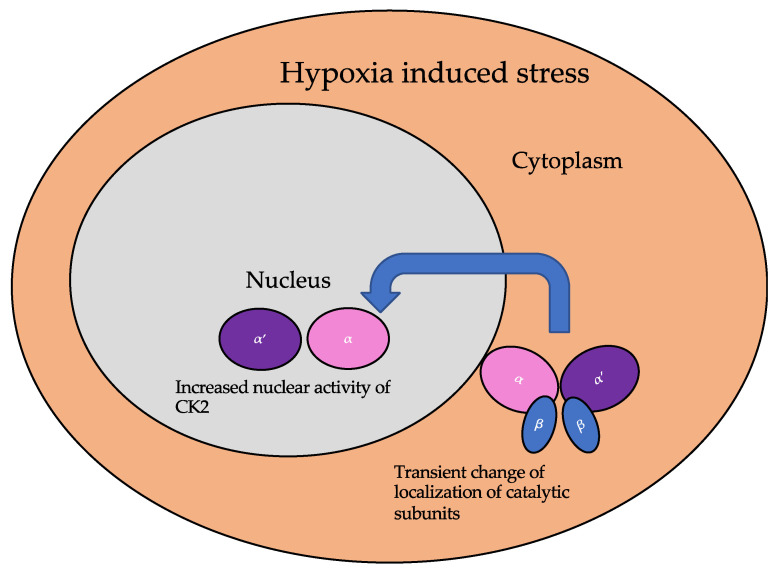
The localization of the catalytic subunits of CK2 is altered during hypoxia. Specifically, these subunits are translocated to the nucleus transiently to aid the cellular response to hypoxia.

The subcellular localization of CK2 determines its regulation, which predominantly includes the cytoplasm, plasma membrane, and nucleus. Interactor proteins and substrates of CK2 are located predominantly in these three cellular compartments [140]. The localization of CK2 varies during cell cycle progression and is sensitive to external stimuli, such as radiation, hypoxia, and stress. Exposure to ionizing radiation (IR) was identified to alter CK2α subunit localization from the cytoplasm to the nucleus in human non-small cell lung cancer cell lines, including H460, A549, and PC9 (Figure 3). The nuclear localization is reported to be transient. In addition, there is an increase in the kinase activity of CK2 during the S phase when cells are exposed to IR. Furthermore, with the pharmacological inhibition of CK2, there is an increase in IR sensitivity [141,142]. This emphasizes the role of CK2 in transmitting radiation stimuli to the nucleus and its involvement in the DNA damage response. Similarly, in M059K human glioma cells, it colocalizes with the IR-induced DNA damage sites [108]. Resistance to IR treatment could be attributed to the dynamic localization of this kinase. During hypoxia, CK2 activity is seen to increase without an increase in its expression, but rather by the transport of catalytic α and α’ subunits to the nucleus (Figure 4). CK2 also plays a role in activating a histone-modifying enzyme, histone deacetylase 2 (HDAC2), in tumors under hypoxic conditions. Here, both catalytic subunits of this kinase are important for the activation of HDAC2 in tumors, and, further, their downregulation impairs the hypoxia-induced activation of histone deacetylase [143]. The nuclear status of CK2 is important for the regulation of several nuclear proteins involved in cell proliferation, survival, and cell death. B23 (also known as nucleophosmin or numatrin) is a nuclear matrix-associated protein that regulates ribosomal RNA (rRNA) synthesis. Its recruitment to specific nucleolar compartments during cellular growth is dependent on its phosphorylation by CK2 [144]. The downregulation of CK2 in prostate cancer cells results in a loss of the nuclear localization of this protein [145].

### 5.2. CK2 in Diseases

CK2 is associated with the etiology of many diseases due to its pleiotropic nature and involvement in almost all cellular process. The diseases associated with the aberrant expression or function of this kinase include cancer, multiple sclerosis, cystic fibrosis, diabetes mellitus, neurological disorders, cardiac hypertrophy, and inflammation [102]. The examples delineated below describe the involvement of CK2 in disease progression based on unique mechanisms specific for the disease. The implication of aberrant CK2 expression and/or function is apparent in several studies.

#### 5.2.1. CK2 in Cancer Progression

CK2 regulates the progression of at least 15 cancer-related proteins including the tumor suppressor p53, histone-modification enzymes such as HDAC1 and HDAC2, and NF-κB subunit RelA [146]. The interaction of additional direct interactors and indirectly regulated proteins remains unclear. There is an upregulation of CK2 during the tumor progression of many cancers and leukemia [147,148]. CK2 itself can be used as a prognostic marker for some cancer types (Table 2) [149]. Proteomic analyses of cell lines indicates that the catalytic CK2α subunit is predominantly expressed in several cancerous cell lines, including U2OS from human osteosarcoma, epidermoid squamous cell carcinoma, brain glioblastoma A549 from lung carcinoma, GAMG from glioblastoma, HEK293 from embryonic kidney cells, HeLa from cervical carcinoma, HepG2 from hepatoma, Jurkat from Acute T cell lymphoma, LnCap from prostate carcinoma, MCF from mammary carcinoma, and RKO from colon carcinoma [131,150,151,152]. Contrasting observations exist for the expression status of CK2. For example, in breast cancer, the nuclear localization of CK2α is emerging as an independent prognostic factor [153]. However, in esophageal cancer, CK2 expression is downregulated [154]. This downregulation resulted from the overexpression of Smad4-mediated transcriptional repression under the influence of the sex-determining region Y-box 2 (SOX-2) [154].

Even further, because a gain-of-function mutation associated with CK2 has yet to be revealed, this enzyme is classified as a tumor-promoting non-oncogene [162]. However, the deletion of *CSNK2B*, which codes for the CK2β subunit, is present in diffuse large B-cell lymphoma (DLBCL) [163]. Additionally, a recent case of the *CSNK2A1* fusion gene is present in a myeloid neoplasm, indicating the diverse roles of CK2 implications in cancer [164].

As CK2 regulates the activation of several proteins required for the cell cycle, the consequences of its overexpression are exacerbated in solid tumors and leukemias [165]. CK2 overexpression and/or overactivity results in the abnormal phosphorylation of its targets. One example is tumor suppressor Ikaros, which is a transcriptional repressor of the genes encoding for Bcl-2-like protein 1 (*BCL2L1*, *PIK3CD*, and *PIKFYVE*) [98,100].

The regulation by second messengers, such as cyclic nucleotides, is important for many oncogenic kinases [166]. However, unlike other kinases except for CK2, there is no regulation by the concentration of second messenger molecules (such as cyclic nucleotides, inositol phosphate, and calcium) or its own phosphorylation status for kinase activity. Instead, the high basal activity of this kinase is demonstrated by its crystallographic structure. The interaction between helix c of the N-terminal region is in tight interaction within the activation loop [167]. Thus, the constitutive activity of CK2 due to its structure adds to the mystery of how this kinase is regulated. The following table classifies general mechanisms by which CK2 is involved in cancer pathogenesis (Table 3) [131].

The central role of CK2 in pathway networks for cell cycle regulation and proliferation makes this protein an interesting pharmacological target for treatment of cancer.

#### 5.2.2. CK2 and Diabetes and Obesity

In pancreatic β cells, a G-protein coupled-receptor muscarinic 3 receptor (M3R) regulates insulin release and homeostasis. CK2 is a negative modulator of M3R. Its phosphorylation of the M3Rs on β-cells inhibits insulin release [171]. Therefore, by phosphorylating the M3Rs, CK2 prevents the release of insulin. However, the abnormal activity of CK2 is linked to diabetes due to its role with these receptors.

Sirtuin1 (SIRT1) deacetylase is a substrate of CK2 and is a regulator for hepatic lipid metabolism. This protein modulates transcription as an energy sensor molecule with its deacetylase function. In diet-induced obesity, CK2 is highly overexpressed and therefore increases the activity of SIRT1. Aberrant phosphorylation of SIRT1 at serine 164 by CK2 inhibits the entry of the former into the nucleus and mildly impairs deacetylase function, thus hampering its regulatory function in hepatocytes, and has been linked to the onset of obesity [172].

#### 5.2.3. CK2 and Heterotrophic Bone Formation

Heterotrophic bone formation in soft tissue due to injury, medication, or as a symptom of another disease can be a painful condition. CK2 phosphorylation stabilizes runt-related transcription factor 2 (RUNX2) by deubiquitylation through the CK2/herpesvirus-associated ubiquitin-specific protease (HAUSP)/RUNX2 pathway. This regulation is important during skeletal development for the fine-tuning of RUNX2, which is a master transcription factor during osteoblastogenesis. However, the overexpression or overactivity of CK2 during heterotrophic bone formation causes ectopic osteoblastogenesis from skeletal stem cells by the same mechanism for RUNX2 stabilization [35].

#### 5.2.4. CK2 and Cardiovascular Diseases

Within the cardiovascular system, CK2α interacts with p27 to prevent its ubiquitination or degradation. Here, the stabilization of p27, which is an inhibitor of cell progression, leads to its accumulation in cytoplasm and the subsequent apoptosis of the cell. Further, CK2α is responsive to external growth stimuli to regulate its interaction with p27. The interaction between CK2α and p27 is interrupted during cardiac hypertrophy [173]. Here, transcriptional reprogramming leading to fetal gene expression causes cardiac hypertrophy. In addition, this reprogramming is related to HDAC2 activity. Specifically, CK2α phosphorylates HDAC2 at serine 394, causing its activation, and leads to cardiac hypertrophy [174].

Patients with cardiac desynchrony are associated with higher risk for morbidity and mortality from heart failure compared to patients with synchronic heart function. Cardiac resynchronization therapy (CRT, pacemaker) is the only known non-pharmaceutical intervention that has long-term effects on this disorder. However, some patients do not respond to this intervention. To study molecular mechanisms for dyssynchronous heart failure (HFdys) and the impact of CRT, proteomic analysis was performed. The comparison of phosphoproteome of HFdys before and after CRT led to the discovery that CK2 signaling is activated during HFdys and that CRT reverses it [175]. The contribution of CK2 signaling in cardiac desynchrony is still an ongoing area of research.

#### 5.2.5. CK2 and Neurodegenerative Disorders

The expression and activity of CK2 in brain cells is involved in neurodegenerative disorders [176]. Here, the overexpression of CK2 is associated with neurodegeneration [177]. Further, there are numerous CK2 substrates associated with brain development and function. In Alzheimer’s disease, CK2 colocalizes with the neurofibrillary tangles. The hyperphosphorylation of the SET protein at serine 9 leads to its improper localization in the cytoplasm caused by the activation of CK2 by tau or β-amyloid (Aβ). The overexpression of CK2 also causes cognitive deficits by impairing synaptic plasticity and synaptogenesis [178]. In Parkinson’s disease (PD), CK2 is localized to Lewy bodies and phosphorylates α-synuclein and synphilin-1 [179]. The treatment of PD using levodopa (L-DOPA) is known to induce L-DOPA-induced dyskinesis (LID). CK2 is important for the regulation of pathways leading to LID. Specifically, CK2α downregulation reduces the severity of LID [176].

Regarding dopaminergic signaling, CK2 is a negative regulator of the D1 receptor (D1R). It binds directly to the Gαs or Gαolf (Golf) subunits, and its knockout leads to the elevated expression of D1R on the plasma membrane. This leads to an increased response to D1 agonists [40]. With the CK2 conditional knockouts in medium spiny neurons, which express dopaminergic receptors, CK2α regulates the D1 signaling pathway in vivo [180].

#### 5.2.6. CK2 and Neurological Disorders

Further, in the neuropsychiatric disorder Tourette syndrome (TS), a mutation in the *SLITRK1* gene is present. The gene product is a membrane-bound protein that regulates synapse formation. The symptoms of TS arise early in the childhood, and affected individuals often suffer from attention-deficit/hyperactivity disorder (ADHD) and obsessive–compulsive disorder (OCD). CK2 phosphorylates the SLITRK1 protein at its 14-3-3 binding site, and a mutation at this phosphorylation site inhibits neurite formation [181].

#### 5.2.7. CK2 in Infectious Diseases

Viral proteins are substrates of CK2. The presence of host kinase phosphorylation sites within these proteins can be useful for the virus to identify host cell status [182]. In the proteome of severe acute respiratory syndrome coronavirus 2 (SARS-CoV-2), a total of 25 phosphorylation sites have been identified. CK2 is one of the main kinases identified to regulate these sites. There is also activation of CK2 signaling observed during SARS-CoV-2 infection [182].

The hepatitis C virus (HCV) nonstructural protein 5A (NS5A) plays an important role in viral particle assembly. Structural studies of the NS5A and CK2 complex using time-resolved nuclear magnetic resonance (NMR) indicate that there are four possible CK2 phosphorylation sites in the NS5A D3 domain [183]. The expression of hepatitis B virus (HBV) is regulated by the Human La protein (hLa). The phosphorylation of hLa at serine 366 by CK2 activates the protein and increases HBV expression. The pharmacological inhibition of CK2 by tetrabromobenzimidazole (TBBz) or a knockdown its gene results in a reduction of HBV expression [184].

In prion diseases, the pathogenic form of the prion protein (Prp) interferes with cellular processes, such as fast axonal transport (FAT), which causes an onset of neurodegenerative disorders. The intracellular accumulation of cellular prion protein (PrP^C^) and full-length PrP (PrP-FL) induces neuronal toxicity, causing severe ataxia in mice, and further, FAT is often inhibited. CK2 inhibits FAT in a similar pattern as in PrP-FL-induced symptoms [48,185,186,187].

#### 5.2.8. Regulation of Immune Response by CK2

The regulation of the immune cell response is targeted for the use of CK2 inhibitors. However, delineating its role during development in the immune response under disease progression has been a challenge. The involvement of CK2 is being investigated during this disease response in ongoing studies.

During *Listeria monocytogenes* (Lm) infection, the adaptive and innate immune responses are involved. In myeloid cell-specific conditional knockout of CK2α in mice, host resistance to Lm infection is significantly increased without affecting myeloid cell development. Myeloid cell recruitment is also seen to be negatively regulated by CK2α [188].

Further, the expression of CK2 is increased in the inflamed mucosa of ulcerative colitis (UC) patients. It is important for maintaining reciprocal balance between Th17 and Treg cells [189]. Interestingly, CK2 activity is reduced, along with an increase in reactive oxygen species (ROS) generation, during acute colitis. NADPH oxidase 1 (NOX1), which is an important regulator of mucosal immunity, is dysregulated during inflammatory bowel disease. Further, CK2 is a suppressor of NOX1 activity [123].

#### 5.2.9. CK2 and Senescence

CK2 activity and expression is downregulated in older animals. Further, the pharmacological inhibition of CK2 induces cellular senescence [190,191]. With artificial downregulation of CK2 by the activity of histone trimethylases, such as histone-lysine N-methyltransferase SUV39H1(SUV39h1), its activity is upregulated in senescence and leads to an increase in tri-methylation at the 9th lysine residue of the histone H3 (H3K9me3) and senescence-associated heterochromatin formation (SAHF). An increase in SAHF leads to the suppression of genes associated with cell cycle progression, such as cyclin D1 [192].

Next, in CK2α knockdown cell lines (MCF-7 and HCT116 cells), the expression of histone demethylases [JmjC domain-containing histone demethylase (JMJD2/KDM4) and lysine-specific demethylase 1 (LSD1/KDM1a)] is downregulated at the translational level. The ectopic expression of these histone demethylases suppressed SAHF and senescence-associated β-galactosidase activity. The effects of the translational downregulation of LSD1 due to the reduction in CK2 activity are complex, since it has non-histone substrates such as p53. The p53/p21Cip1/WAF1 pathway, which is an upstream regulator of SUV39h1 and H3K9me3, is also upregulated in CK2α knockdown cells. [193,194]. Therefore, this pathway is important for CK2-downregulation-mediated senescence.

## 6. Clinical Applications of CK2

**Figure 5:**
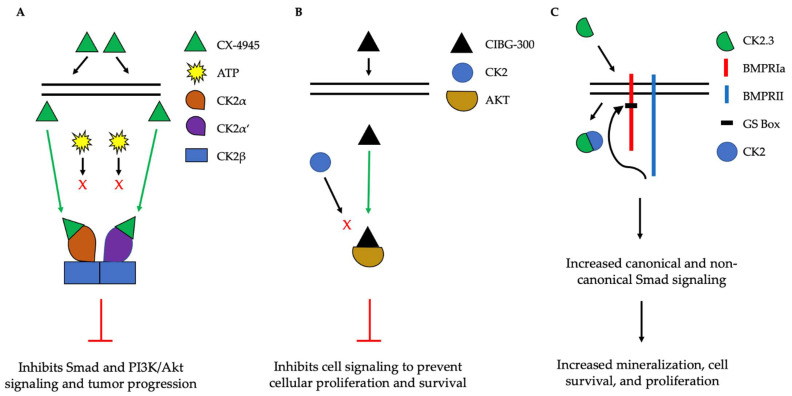
Simplified schematic demonstrating three therapeutics targeting the activity of CK2. (**A**). CX-4945 is a competitive ATP inhibitor that binds preferentially to ATP-pocket domains. Here, it binds to both CK2α and CK2α’, thereby preventing ATP from binding and activating CK2. CX-4945 has prevented tumor progression by inhibiting signaling pathways such as Smad and PI3K/AKT. (**B**). CIBG-300 functions by binding to conserved phosphorylation sequences on the substrates of CK2, such as Akt. This association prevents the phosphorylation of these substrates by CK2, leading to decreased cell signaling, proliferation, and survival. (**C**). CK2.3 is uptaken by cells and binds to CK2, therefore preventing its association with BMPRIa. As a result, BMPRII can phosphorylate BMPRIa, leading to the downstream activation of signaling pathways that induce mineralization and cell survival.

The implication of CK2 in the progression of many diseases has led to its popularity as a therapeutic target. Specifically, researchers have attempted to prevent the kinase activity of CK2, thereby to prevent pathological activity. In cells, CK2 contains pocket domains for adenosine triphosphate (ATP)-binding [195]. The pocket domain is essential for the activation and subsequent enzymatic activity of CK2. Therefore, blocking this domain may deactivate CK2. One such inhibitor under investigation is CX-4945, known as silmitasertib in the clinic. CX-4945 is currently in clinical trials to treat cancer and is being tested as an oral administration to patients to limit the activity of CK2. Specifically, CX-4945 functions as an ATP-competitive inhibitor, leading to cell cycle arrest and apoptosis [196]. Here, CX-4945 inhibits the activity of both CK2α and CK2α’ by binding to the ATP-binding site of these subunits with a higher affinity than ATP [195,197,198,199]. This competitive binding leads to the decreased expression of CK2 and its subsequent signaling, such as Smad and PI3K/Akt pathways [196,199] (Figure 5A). This decreased activation of these pathways by CX-4945 has inhibited the progressions of several cancers, including gastric cancer, renal cancer, hematological cancer, cholangiocarcinoma, basal cell carcinoma, and medulla blastoma [24,197,200,201]. CX-4945 is a promising therapeutic agent and has displayed minimal side-effects, such as diarrhea, nausea, and anemia, suggesting its role as a safe treatment [201,202].

Another CK2 inhibitor, known as CIBG-300, is also currently in clinical trials as a therapeutic to treat cancer [106,203]. This inhibitor binds specifically to CK2 to prevent cellular proliferation; leads to decreased cell adhesion and migration; and promotes the apoptosis of the cells, specifically in cervical cancer, breast cancer, and colorectal cancer, both in vivo and in vitro [203,204,205,206,207,208,209,210]. Here, CIBG-300 functions by binding to the conserved phosphorylation sequences of CK2 substrates [207,211]. Subsequently, CK2 is unable to phosphorylate its downstream target; CIBG-300 thereby prevents the activity of various proteins involved in cancer progression and inhibits angiogenesis [207,208,209,212,213]. More interestingly, CIBG-300 inhibits the activation of Akt, PI3K, PTEN, and NF-κB signaling pathways, therefore decreasing cell proliferation and survival [95,214,215] (Figure 5B). Further, CIBG-300 treatment leads to limited side-effects including edema, hot flashes, tachycardia, lower abdominal pain, and bleeding [213]. Thus, this potential therapeutic has been efficient in clinical trials and holds promise to treat a wide array of cancers.

In addition, inhibiting the enzymatic activity of CK2 can be used to treat other disorders, such as osteoporosis (OP) and osteoarthritis (OA). CK2 is expressed in bone cells, such as osteoblasts and osteoclasts. Within these cells, CK2 is bound to the intracellular domain of BMPRIa at its phosphorylation sites. As a result, the BMP-signaling pathway is inactive until an extracellular ligand, such as BMP-2, binds to BMPRIa to cause a conformational change of BMPRIa and release CK2. This release allows BMPRII to phosphorylate BMPRIa, leading to the downstream activation of Smad and non-Smad signaling to increase bone and cartilage formation. However, as humans age, bone mineral density and cartilage decrease; these changes lead to osteoporosis and osteoarthritis, even in the absence of extracellular ligands, respectfully. The interaction between CK2 and BMPRIa can be disrupted intracellularly to increase Smad and non-Smad signaling. Here, the Nohe lab constructed mimetic peptides named CK2.1, 2.2, and 2.3, each of which contain a phosphorylation site sequence of BMPRIa. This sequence is flanked by other amino acids, and the peptides contain the antennapedia homeodomain for cellular uptake. Once endocytosed by cells, CK2.X binds to CK2 and prevents it from associating with BMPRIa. CK2.1 (phosphorylation site SYED) leads to chondrogenesis; CK2.2 (phosphorylation site SLYD) leads to adipogenesis, and CK2.3 (SLKD) leads to osteogenesis. Further, CK2.3 upregulates signaling and leads to increased mineralization in primary osteoblasts isolated from OP patients. Further, this peptide enhances bone mineralization in both in vivo and in vitro models in the absence of BMP-2 (Figure 5C). These peptides, along with the BMP-signaling pathway, may be useful in treating bone disorders such as OP.

## 7. Discussion

This review aimed to describe the current roles of CK2 in development and adulthood, as well as describing its role in signaling pathways and disease progression. CK2 is essential for a myriad of developmental processes and the regulation of signaling pathways throughout adulthood. CK2 is involved in neurogenesis, cardiogenesis, spermatogenesis, and limb formation. Further, this enzyme has over 200 known phosphorylation substrates and is involved in pathways including cellular proliferation and survival, osteogenesis, angiogenesis, and chondrogenesis. However, due to the ubiquitous expression and constitutive activity of CK2, it has been well documented to be involved in the progression of many cancers, bone disorders, neurological and neurodegenerative disorders, immune disorders, and infectious diseases. Further, as the role of CK2 in various diseases is established, it has also become a popular target to treat the aforementioned disorders. With the novel findings about the role of the individual subunits of CK2 during disease progression, the targeting of CK2 activity may become more effective. Further, studies with its cell-lineage-specific function in contrast to its ubiquitous expression and constitutive activity can identify specific pathways for therapeutics to target. Specifically, two peptide-derived therapeutics are currently in clinical trials, known as CX-4945 and CIBG-300. These peptides have been successful in inhibiting the proliferation and progression of cancers and tumors. Further, other peptides currently being investigated in research are CK2.1 and CK2.3, which may be utilized to treat osteoarthritis and osteoporosis, respectively. Therefore, targeting CK2 and further elucidating its role in disease progression may be useful in treating many different disorders.

## Figures and Tables

**Table 1:** Phenotypes associated with CK2-specific subunit knockouts.

Subunit	Organism/Cell Type	Associated Phenotype/Molecular Effect	References
**CK2α**	***Schizosaccharomyces pombe***	Defects in polarized cell growth	[36]
**CK2α**	**Mouse B cells**	Aberrant accumulation of marginal zone B cells	[38]
**CK2α**	**C2C12 cells**	Impaired myotube formation in comparison to WT, reduction in CK2β expression	[39]
**CK2α**	**Medium spiny neurons**	Altered dopamine signaling	[40]
**CK2α**’	**C2C12 cells**	Reduction in the number and size of myotubes, defective skeletal muscle cell fusion	[39]
**CK2α**’	**GN11 cells**	Decrease in cell migration, increased cell adhesion and fibronectin expression, activation of focal adhesion molecules: focal adhesion kinase (FAK), paxicillin, AKT activation, glycogen synthase kinase 3 beta (GSK3β) activation	[41]
**CK2β**	**C2C12 cells**	Decreased expression of early muscle differentiation factor myoD	[39]
**CK2β**	**GN11 cells**	Decrease in cell migration and adhesion, weak activation of Fax, paxillin, and AKT, general actin depolymerization	[41]
**CK2β**	**HK-2 cells**	Decreased AKT S129 (activating) phosphorylation, decreased eukaryotic translation initiation factor 2 subunit beta (elf2β) phosphorylation, deceases in phosphatase and tensin homolog (PTEN) expression	[42]
**CK2β**	**Mouse**	Age-dependent weakness and decreased grip strength	[43]
**CK2β**	**Mouse muscle skeletal fibers**	Changes in oxidative metabolism, dysfunctional mitochondria	[44]

**Table 2:** CK2-subunit overexpression is a prognostic marker for several types of tumors.

Cancer Type	Form of CK2	References
Acute myeloid leukemia	CK2α	[155]
Primary hepatocellular carcinoma	CK2α	[156]
Gastric carcinoma	CK2β	[157]
Squamous cell carcinoma of the head and neck	CK2	[158]
Renal cell carcinoma	CK2α	[159]
Colorectal cancer	CK2α	[160]
Breast cancer	CK2α	[161]

**Table 3:** The involvement of CK2 in tumorigenic mechanisms.

Mechanism	References
Sustained activation of growth signal proteins and stabilization of tumor suppressors including the phosphorylation of PTEN (stabilizing); the modulation of mTORC1 and MAP kinase pathways; the activation of NF-κB, signal transducer and activator of transcription 3 (STAT3), and AKT/FOXO signaling pathways	[101,108,168,169]
Anti-apoptotic function to antagonize death-receptor-induced apoptosis	[149]
Multiple-drug-resistance phenotype	[170]
Induction of the Warburg effect	[102]
Modulation of mitochondrial function including an increase in lactate dehydrogenase, pyruvate kinase1 overexpression, and hypoxia inducible factor-1 (HIF-1) activation	[102]
Activating metastases	[47]
Dysregulation of DNA repair mechanisms	[165]

## Data Availability

Not applicable.
